# Development and validation of a GC × GC-ToFMS method for the quantification of pesticides in environmental waters

**DOI:** 10.1007/s00216-023-04686-8

**Published:** 2023-04-24

**Authors:** Monica Romagnoli, Andrea Scarparo, Martina Catani, Biagio Giannì, Luisa Pasti, Alberto Cavazzini, Flavio A. Franchina

**Affiliations:** 1 Department of Chemical, Pharmaceutical, and Agricultural Sciences, University of Ferrara, via L. Borsari 46, 44121 Ferrara, Italy; 2 Agenzia Regionale per la Protezione dell’Ambiente – ARPAV, via Lissa, 30174 Mestre, Italy; 3 Department of Environmental and Prevention Sciences, University of Ferrara, Via L. Borsari 46, 44121 Ferrara, Italy

**Keywords:** Pesticides, Comprehensive two-dimensional gas chromatography, Mass spectrometry, Method validation, Water analysis

## Abstract

Water is a fundamental resource for living things, which is why its control is necessary. The widespread use of pesticides for agricultural and non-agricultural purposes has resulted in the presence of their residues in surface water and groundwater resources. Their presence in water is regulated through different directives, such as the Groundwater Directive, the Drinking Water Directive, and the Water Framework Directive, modified later several times, setting a maximum concentration of 0.1 µg.L^−1^ for individual pesticides and their degradation products, and 0.5 µg.L^−1^ for total pesticide residues present in a sample. There are different kinds of pesticides (e.g., organophosphorus and organochlorine pesticides, triazines, chloroacetamides, triazoles, (thio)carbamates) that have diverse chemical structures. Their determination and monitoring in a single analytical procedure are possible through multiresidue methods. In this study, 53 pesticides belonging to different chemical classes and their metabolites were selected based on their local occurrence and investigated in surface water and groundwater from agricultural areas susceptible to pesticide contamination. The methodology consisted of a classical solid-phase extraction (SPE) for the purification and enrichment of the pesticides, with a subsequent analysis in multidimensional gas chromatography coupled to mass spectrometry (GC×GC-MS). The quantification method was validated according to the Eurachem Guide in terms of linearity, precision, accuracy, limit of detection, and limit of quantification. After validation, the method was applied to 34 real-world water samples, and the results were compared with those obtained by a GC-QMS routine method.

**Supplementary Information:**

The online version contains supplementary material available at 10.1007/s00216-023-04686-8.

## Introduction

Pesticides are chemical compounds that are used in agriculture to prevent or reduce problems caused by pests [1]. In 2019, the European Commission estimated that there were 463 type of pesticides whose use has been approved, 667 not approved and 48 that are under review by the same organization, for a total of 1331 type of existing pesticides [1, 2].

Because of the great diversity of these compounds, they can be classified in different ways: based on the target organism on which they act (such as herbicides, bactericides, fungicides), the entry mode on the pests, the chemical structure (e.g., organochlorine, carbamate, organophosphorus), and the degree of risk to public health [1, 3]. The main problem with the use of these compounds is that they have an impact on human health and the environment[3, 4]. This phenomenon is amplified in developing countries due to the use of pesticides which are unpatented, cheaper, more toxic, and persistent in the environment [5].

For this reason, the Stockholm Convention on Persistent Organic Pollutants (POPs) established chemicals whose production and use should be eliminated or restricted. In the first meeting, in 2001, 12 POPs were listed and 9 of which were pesticides; in 2009, during the second meeting, 16 new POPs were added to the list and 7 of which were pesticides [6].

An important problem related to the use of pesticides is that they are found in water, both surface and groundwater, modifying its quality and causing harm to all type of life, not just humans [7]. The pollution of water by pesticides is due to agricultural activities, urban use, and pesticide production factories [8]. During pest treatment, these substances are sprayed on plants, but a small amount reaches the soil and then can diffuse into water bodies through different pathways: spray drift, runoff and wind erosion events, leaching and vaporization, and subsequent dry deposition [9].

To safeguard human health, waters of water bodies must undergo controls before they can become potable. For this reason, the European Union set threshold values of pesticides and their degradation products in surface and groundwater of 0.1 μg.L^−1^ and 0.5 μg.L^−1^ for individual and total sum, respectively [10–12].

For their monitoring, after water sampling, the purification and enrichment through solid-phase extraction (SPE) represent the method of choice in most routine laboratories, and the resulting extracts can then be used for the following chromatographic analysis [13, 14]. Gas chromatographic (GC) separation can cover the volatilizable pesticides, often coupled to selective detectors like the electron capture detector (ECD), nitrogen/sulfur chemiluminescence detector (NCD/SCD), or mass selective detector (MSD) [15, 16]. Among them, mass spectrometry gives the ultimate selectivity to the hyphenated method, thanks to its direct information on the analyte structure. The instrumental technological progress makes nowadays available a variety of robust and reliable mass analyzer (i.e., quadrupole (QMS), triple quadrupole, time-of-flight (ToFMS), Orbitrap) [17]. Comprehensive two-dimensional gas chromatography (or GC×GC) represents a technical advancement nowadays available [18] which is more and more applied, also for target phytosanitary product analysis [19–25].

The aim of this study is the development and validation a GC×GC-ToFMS method for the quantification of 53 pesticides in surface and groundwater. In addition, a comparison of the results on 34 real-world samples between the GC×GC-ToFMS and a GC-QMS routine method used by the regional environmental control agency was performed.

## Materials and methods

### Chemicals and solvents

The solvents used were ethyl acetate and methanol of analytical grade and obtained from Merck (Darmstadt, Germany).

The chemicals used were three custom mixes obtained by o2si Smart Solution (North Charleston, SC, USA). Mix A contained alachlor, ametryne, atrazine, bladex, chlorpyrifos-methyl, chlorpyrifos-ethyl, o,pʹ-DDT, p,pʹ-DDT, desethyl atrazine, malathion, metolachlor, molinate, oxadiazon, pendimethalin, prometon, prometryn, simazine, terbuthylazine, desethyl terbuthylazine, terbutryn, and vinclozolin in acetone each at a concentration of 100 mg.L^−1^. Mix B contained δ-BHC, captan, chlorfenvinphos, p,pʹ-DDD, p,pʹ-DDE, dimethenamid, dimethoate, α-endosulfan, β-endosulfan, endosulfan sulfate, ethofumesate, flufenacet, folpet, procymidone, propanil, propyzamide, and caffeine in methanol each at 100 mg.L^−1^. Mix C contained aldrin, α-BHC, β-BHC, γ-BHC, chlordane, dieldrin, endrin, heptachlor, hexachlorobenzene, isodrin, pentachlorobenzene, and trifluralin in methanol at 100 mg.L^−1^. Metazachlor (100 mg.L^−1^ in acetonitrile) was obtained by Dr. Ehrenstorfer (Augsburg, Germany), and heptachlor epoxide (1000 mg.L^−1^ in methanol) was obtained by A2S Analytical Standard Solutions (Saint Jean d’Illac, France). An instrumental and a procedural internal standard used were, respectively, azobenzene (I-IS) in acetone at a concentration of 100 μg.mL^−1^ and atrazine-d_5_ (P-IS) in ethyl acetate at 100 μg.mL^−1^ (both from A2S Analytical Standard Solutions). These were added prior the GC injection and prior the extraction, respectively.

### Samples and analytes extraction

In this study, after the method validation, 34 real-world samples, collected locally, were analyzed in duplicate and quantified. Seven were groundwaters, while the remaining 27 samples were surface waters.

Solid-phase extraction was used to extract the target analytes from samples. Briefly, 250 μL of the surrogate aka procedural internal standard P-IS (atrazine-d_5_, 100 μg.L^−1^ in methanol) was added to 500 mL of starting sample. This solution was loaded to OASIS HLB 6 cc 200 mg cartridge, previously conditioned and equilibrated with 4 mL of ethyl acetate, 4 mL of methanol, and 5 mL of water. The cartridge was dried by a N_2_ stream, and the elution was performed through 2.5 mL of ethyl acetate. The dried residue was dissolved in 250 μL of ethyl acetate containing 250 μg.L^−1^ of instrumental internal standard I-IS (azobenzene).

Similarly, the analytical curve levels were prepared diluting in ethyl acetate the mixture of standards, including P-IS. After, an aliquot of 250 µL was N_2_-dried, and the residue was dissolved in 250 µL of ethyl acetate containing the I-IS (250 µg.L^−1^). The actual concentration to build the calibration curve was 20, 50, 100, 150, 200, and 300 μg.L^−1^, corresponding to 0.01, 0.025, 0.05, 0.075, 0.1, and 0.15 μg.L^−1^, after dilution correction. Blank sample was also regularly carried out to exclude preparative and instrumental carryover.

### Instrumental experimental conditions

The development and validation of the GC×GC-MS method were conducted on a Pegasus BT 4D (LECO Corporation, Mönchengladbach, Germany) equipped with an Agilent 8890 GC and an Automatic Liquid Sampler (Agilent Technologies, Santa Clara, CA, USA). The chromatographic columns were a 30 m × 0.25 mm × 0.25 μm *d*_*f*_ Rxi-5SilMS as the first dimension and a 2 m × 0.25 mm × 0.25 μm d_*f*_ Rxi-17SilMS as the second dimension (both from Restek Corporation, Bellefonte, PA, USA). The injections were performed in split mode (1:10), injection volume of 2 μL, inlet temperature of 250 °C; the carrier gas was helium used in constant flow mode (1.30 mL.min^−1^). The oven temperature program was 140 °C (held 1 min), then ramped at 6 °C.min^−1^ to 270 °C, and finally ramped at 20 °C.min^−1^ to 320 °C (held 2 min). Temperature offsets for the secondary oven and for the quad-jet dual-stage cryogenic modulator were set at + 25 °C and + 15 °C, respectively. A 2.6-s modulation period was used. A mass range from 40 to 500 m*/z* was collected with an acquisition frequency of 150 Hz. An acquisition delay of 300 s was used. The transfer line and ion source temperatures were both set at 250 °C.

Data were collected and analyzed using ChromaTOF® BT software version 5.54.80.0.1131 (LECO Corporation). The signal-to-noise threshold was set at 100, and the NIST20 mass spectral library was used for putative identification using a spectral similarity > 80%. A calibration curve level of pesticide mix was used to find retention times of targeted compounds. Compounds were searched using the target search function, in which the ^1^D and ^2^D retention times (tolerance of 0.4 min and 0.7 s, respectively) and the exact masses of characteristic ions (tolerance 0.10 Da) were set. Peak integration was carried out considering extracted ions (the quantifier ions are reported in Table 1), and the areas were exported to Microsoft Excel for further statistical elaboration.

**Table 1:** List of the target pesticides, along with the quantifier ion and the method validation figure of merit (linearity range, coefficient of determination, LOD, and LOQ)

Peak #	Analyte	Type of pesticide-chemical class	Quantifier ion (*m/z*)	Linearity range (µg.L^−1^)	Calibration curve	*R*^2^	LOD (µg.L^−1^)	LOQ (µg.L^−1^)
1	Benzene, pentachloro-	Fungicide-organochlorine	249.8	0.01–0.15	*y* = 0.0026*x* − 0.0031	0.9969	0.0019	0.0062
2	Molinate	Herbicide-thiocarbamate	126.1	0.01–0.15	*y* = 0.0015*x* + 0.0045	0.9991	0.0011	0.0037
3	Desethylatrazine	Herbicide-chlorotriazine	172.0	0.01–0.15	*y* = 0.0007*x* − 0.0023	0.9988	0.0011	0.0035
4	Trifluralin	Herbicide-dinitroaniline	306.1	0.01–0.15	*y* = 0.0002*x* − 0.0010	0.9986	0.0015	0.0048
5	Desethylterbuthylazine	Herbicide-chlorotriazine	186.1	0.01–0.15	*y* = 0.0008*x* − 0.0034	0.9981	0.0008	0.0026
6	α-BHC	Insecticide-organochlorine	180.9	0.01–0.15	*y* = 0.0013*x* − 0.0019	0.9991	0.0007	0.0024
7	Benzene, hexachloro-	Fungicide-organochlorine	283.8	0.01–0.15	*y* = 0.0017*x* + 0.0094	0.9966	0.0021	0.0070
8	Dimethoate	Insecticide-organophosphate	87.0	0.01–0.15	*y* = 0.0009*x* + 0.0116	0.9998	0.0017	0.0057
9	Simazine	Herbicide-chlorotriazine	201.1	0.01–0.15	*y* = 0.0002*x* − 0.0013	0.9977	0.0014	0.0047
10	Atrazine	Herbicide-chlorotriazine	200.1	0.025–0.15	*y* = 0.0004*x* − 0.0029	0.9982	0.0010	0.0033
11	β-BHC	Insecticide-organochlorine	110.9	0.01–0.10	*y* = 0.0011*x* + 0.0074	0.9994	0.0007	0.0022
12	Lindane	Insecticide-organochlorine	180.9	0.05–0.15	*y* = 0.0011*x* − 0.0061	0.9987	0.0007	0.0022
13	Terbuthylazine	Herbicide-chlorotriazine	214.1	0.025–0.15	*y* = 0.0005*x* − 0.0045	0.9980	0.0009	0.0030
14	Propyzamide	Herbicide-benzamide	172.9	0.01–0.10	*y* = 0.0008*x* − 0.0015	0.9983	0.0008	0.0026
15	δ-BHC	Insecticide-organochlorine	180.9	0.05–0.15	*y* = 0.0009*x* − 0.0066	0.9989	0.0006	0.0018
16	Caffeine	Insecticide-xanthine	194.1	0.01–0.15	*y* = 0.0006*x* + 0.0046	0.9963	0.0021	0.0069
17	Propanil	Herbicide-chloroacetanilide	160.9	0.01–0.10	*y* = 0.0005*x* + 0.0072	0.9919	0.0017	0.0055
18	Dimethenamid	Herbicide-chloroacetamide	154.1	0.05–0.15	*y* = 0.0011*x* − 0.0060	0.9989	0.0006	0.0020
19	Chlorpyrifos-methyl	Insecticide-organophosphate	124.9	0.05–0.15	*y* = 0.0008*x* + 0.0006	0.9992	0.0013	0.0043
20	Alachlor	Herbicide-chloroacetanilide	45.1	0.01–0.15	*y* = 0.0014*x* + 0.0092	0.9998	0.0008	0.0027
21	Heptachlor	Insecticide-organochlorine	100.0	0.01–0.15	*y* = 0.001*x* + 0.0068	0.9992	0.0007	0.0022
22	Terbutryn	Herbicide-triazine	226.1	0.05–0.15	*y* = 0.0002*x* − 0.0041	0.9979	0.0011	0.0036
23	Aldrin	Insecticide-organochlorine	66.1	0.01–0.10	*y* = 0.0011*x* + 0.0246	0.9988	0.0012	0.0040
24	Metolachlor	Herbicide-chloroacetanilide	162.1	0.01–0.10	*y* = 0.0010*x* + 0.0003	0.9991	0.0003	0.0011
25	Chlorpyrifos-ethyl	Insecticide-organophosphate	96.9	0.01–0.15	*y* = 0.0007*x* + 0.0107	0.9991	0.0016	0.0052
26	Isodrin	Insecticide-organochlorine	192.9	0.01–0.10	*y* = 0.0005*x* + 0.0034	0.9991	0.0010	0.0032
27	Metazachlor	Herbicide-organochlorine	81.0	0.01–0.15	*y* = 0.001*x* + 0.0181	0.9978	0.0009	0.0031
28	Pendimethalin	Herbicide-dinitroaniline	252.1	0.01–0.15	*y* = 0.002*x* − 0.0006	0.9990	0.0007	0.0021
29	Heptachlor epoxide	Insecticide-organochlorine	81.0	0.01–0.15	*y* = 0.0005*x* + 0.0093	0.9930	0.0014	0.0046
30	Chlorfenvinphos	Insecticide-organophosphate	266.9	0.025–0.10	*y* = 0.0001*x* − 0.0005	0.9988	0.0013	0.0043
31	α-Endosulfan	Insecticide-organochlorine	63.9	0.01–0.15	*y* = 0.0011*x* + 0.0242	0.9998	0.0017	0.0057
32	p,pʹ-DDE	Insecticide-organochlorine	246.0	0.05–0.15	*y* = 0.0008*x* − 0.0102	0.9972	0.0009	0.0031
33	Oxadiazon	Herbicide-organochlorine	174.9	0.05–0.15	*y* = 0.0004*x* − 0.0057	0.9972	0.0011	0.0035
34	Dieldrin	Insecticide-organochlorine	79.1	0.01–0.15	*y* = 0.001*x* + 0.0157	0.9970	0.0007	0.0023
35	Endrin	Insecticide-organochlorine	81.0	0.05–0.15	*y* = 0.0003*x* + 0.0079	0.9983	0.0009	0.0031
36	β-Endosulfan	Insecticide-organochlorine	63.9	0.01–0.15	*y* = 0.0012*x* + 0.034	0.9980	0.0014	0.0046
37	p,pʹ-DDD	Insecticide-organochlorine	235.0	0.05–0.15	*y* = 0.0009*x* − 0.0113	0.9979	0.0009	0.0028
38	o,pʹ-DDT	Insecticide-organochlorine	235.0	0.01–0.15	*y* = 0.0008*x* − 0.0037	0.9984	0.0008	0.0027
39	p,pʹ-DDT	Insecticide-organochlorine	235.0	0.025–0.15	*y* = 0.0007*x* − 0.0057	0.9986	0.0007	0.0021
I	Prometon	Herbicide-triazine	58.1	0.01–0.15	*y* = 0.0006*x* + 0.0093	0.9998	0.0016	0.0053
II	Vinclozolin	Fungicide-dicarboximide	186.9	0.05–0.15	*y* = 0.0003*x* − 0.0044	0.9968	0.0007	0.0023
III	Ametryn	Herbicide-triazine	68.0	0.01–0.15	*y* = 0.0003*x* + 0.0111	0.9978	0.0014	0.0047
IV	Prometryn	Herbicide-triazine	184.1	0.05–0.15	*y* = 0.0003*x* − 0.0038	0.9987	0.0014	0.0047
V	Ethofumesate	Herbicide-benzofuranyl alkyl sulfonate	161.1	0.01–0.10	*y* = 0.0004*x* + 0.0047	0.9993	0.0022	0.0072
VI	Malathion	Insecticide/miticide-organophosphate	173.1	0.01–0.10	*y* = 0.0003*x* − 0.0004	0.9992	0.0007	0.0024
VII	Bladex	Herbicide-triazine	68	0.05–0.15	*y* = 0.0004*x* + 0.0090	0.9985	0.0017	0.0056
VIII	Flufenacet	Herbicide-oxyacetanilide	151.1	0.01–0.15	*y* = 0.0004*x* + 0.0011	0.9997	0.0008	0.0027
IX	Captan	Fungicide-phthalimide	79.1	0.05–0.15	*y* = 0.0010*x* + 0.0368	0.9941	0.0014	0.0045
X	Folpet	Fungicide-thiophthalimide	104.0	0.01–0.15	*y* = 0.0003*x* + 0.0140	0.9988	0.0017	0.0056
XI	Procymidone	Fungicide-dicarboximide	96.1	0.01–0.15	*y* = 0.0014*x* + 0.0111	0.9998	0.0015	0.0050
XII	Trans-Chlordane	Insecticide-organochlorine	372.8	0.05–0.15	*y* = 4.914^10^−5^*x* − 0.0016	0.9943	0.0014	0.0047
XIII	Cis-Chlordane	Insecticide-organochlorine	372.8	0.05–0.15	*y* = 3.497^10^−5^*x* − 0.0011	0.9960	0.0012	0.0040
XIV	Endosulfan sulfate	Insecticide-organochlorine	271.8	0.05–0.15	*y* = 0.0002*x* – 0.004	0.9981	0.0009	0.0029

The original routine 1D method relied on a GC Clarus 680 coupled to a single quadrupole mass spectrometer SQ8T (Perkin Elmer, Waltham, MA, USA). The chromatographic column was a 30 m × 0.25 mm × 0.25 μm d_*f*_ J&W DB-5MS UI (Agilent Technologies). The carrier gas was helium used in constant flow mode of 1 mL.min^−1^. The injections were performed in splitless mode, with an injection volume of 2 μL and inlet temperature of 250 °C. The oven temperature program was 50 °C (held 2 min), then ramped at 25 °C.min^−1^ to 150 °C (held 1 min), and subsequently ramped at 4 °C.min^−1^ to 260 °C (held 8 min). The mass acquisition was performed in SIM mode, detecting 3 specific ions per compound (one quantifier and two qualifiers, not reported) and using 180 s of acquisition delay.

### Validation strategy and figures of merit

The GC×GC-MS developed method has been validated in terms of linearity, sensitivity, trueness, precision, and extraction recovery following Eurachem Guide [26]. The analytical curves were constructed by six calibration levels (corresponding to final concentrations of 0.01, 0.025, 0.05, 0.075, 0.1, and 0.15 μg.L^−1^) analyzed for a total of nine times in 3 different days, as suggested in [27]. The least squares method was applied to estimate the regression lines, and linearity was further assessed using Mandel’s fitting test (*p* < 0.05).

Precision was evaluated at the lowest, middle, and highest calibration levels (0.01, 0.075, and 0.15 μg.L^−1^), both intra and inter-day, as coefficient of variation (CV):

$$CV\%=\frac{s}{\overline{{~}^{{A}_{S}}\!\left/ \!{~}_{{A}_{IS}}\right.}}\bullet 100$$

where *s* is the standard deviation and *A*_*S*_ and *A*_*IS*_ are the areas of analyte and internal standard, respectively.

Trueness was assessed on two levels (0.03 and 0.125 μg.L^−1^, *n* = 3) by calculating the bias as

$$bias\%=\left(\frac{{\overline{x} }_{exp}}{{x}_{real}}-1\right)\bullet 100$$

where ${\overline{x} }_{exp}$ is the experimental average concentration and ${x}_{real}$ the theoretical concentration.

Moreover, accuracy was evaluated with the same analyses of the calibration curve both inter- and intra-days, as reported by Alladio et al. [27, 28]. In this approach, for the intra-day study, two repetitions of a day were used to compute the calibration curve, and the last was interpolated to obtain the concentrations; this operation was performed to calculate the concentrations of all three repetitions. The concentrations calculated in this way were averaged, and the bias was calculated as follows:

$$bias\%=\left(1-\frac{{x}_{real}}{{\overline{x} }_{exp}}\right)\bullet 100$$

For the inter-day, the same methodology was used by computing the calibration curve with the six repetitions of 2 days and calculating the concentrations of the data-points from the third day.

The limit of detection (LOD) and the limit of quantification (LOQ) were estimated as 3 ${\mathrm{s}}_{0}^{\mathrm{^{\prime}}}$ and 10 ${\mathrm{s}}_{0}^{\mathrm{^{\prime}}}$, respectively [26].

Extraction recovery was evaluated on the lowest and highest concentration levels. For each target compound, the normalized area in the blank spiked before the extraction process ((A_S_⁄A_IS_)_before_) was considered and compared to a blank fraction that was spiked after the SPE process ((A_S_⁄A_IS_)_after_) [28]:

$$ER\%=\frac{{\overline{{\left({~}^{{A}_{S}}\!\left/ \!{~}_{{A}_{IS}}\right.\right)}}}_{before}}{{\overline{{\left({~}^{{A}_{S}}\!\left/ \!{~}_{{A}_{IS}}\right.\right)}}}_{after}}\bullet 100$$

## Results and discussion

### GC×GC-ToFMS method validation

Initially, the GC×GC method was developed from the 1D GC analog, which was used routinely for the monitoring of 39 pesticides in surface water and groundwater, according to the legislation and the research protocols by the Italian national institute of health; specifically, the protocol for the multiresidual determination of phytosanitary compounds in water samples relies on traditional chromatographic methods coupled to mass spectrometry or other selective detectors, after SPE purification and enrichment [14].

For the GC×GC method, the same ^1^D non-polar column (5% diphenyl) was maintained (non-polar/polar column set), and a faster temperature program was used, reducing the analysis time to 26 min (40% faster compared to the 1D GC-QMS routine approach). More specifically, all the target analytes were eluted within 18 min separation in the GC×GC method.

**Fig. 1:**
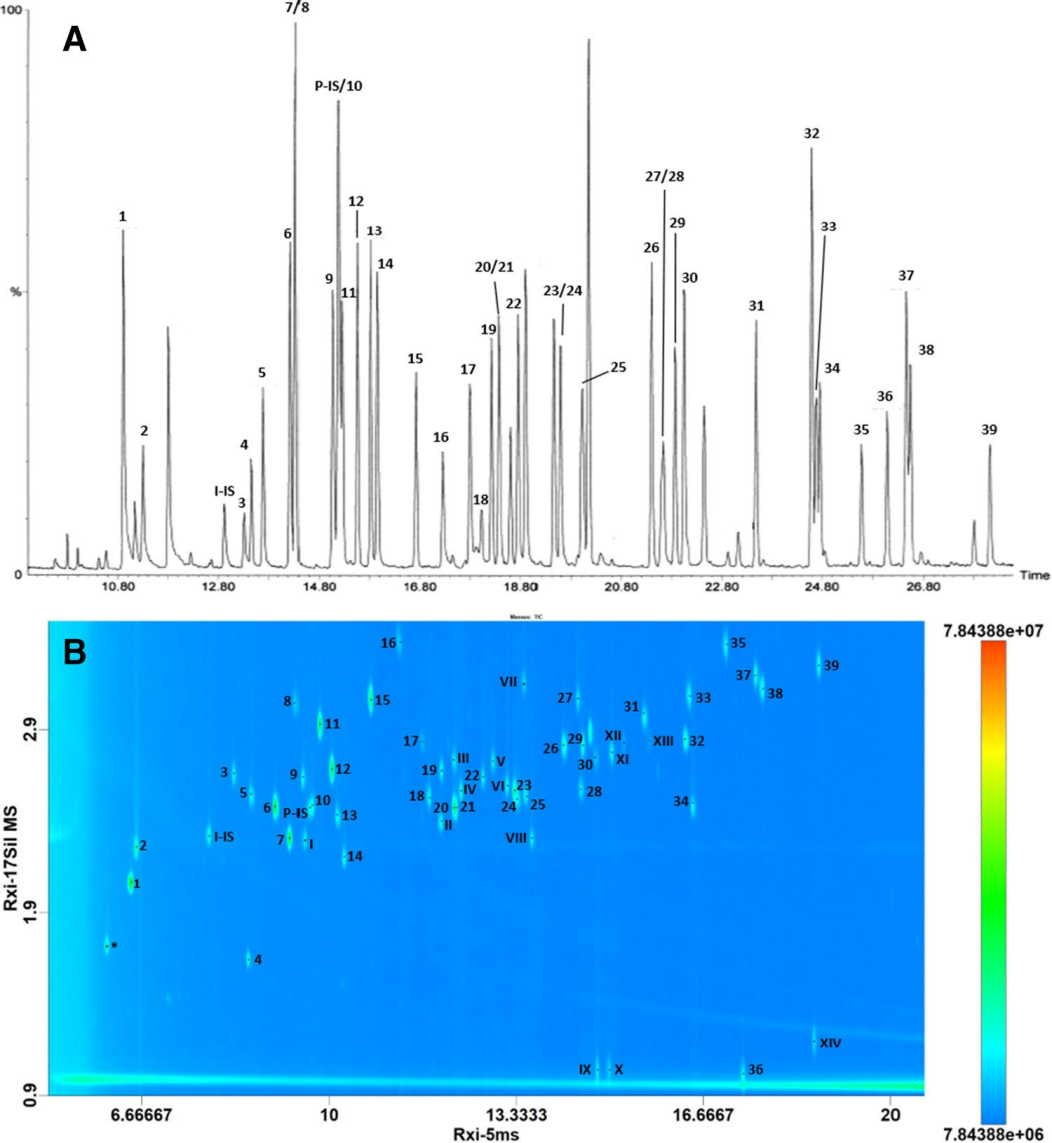
**A** Zoom of the GC-QMS chromatogram of the standard pesticides mix (reconstructed ion chromatogram) at 500 μg.L^−1^. **B** Zoom of GC×GC-ToFMS analysis chromatogram of standard mix (total ion chromatogram) at 200 μg.L^−1^ with internal standards (P-IS and I-IS). For peak number, refer to Table 1; the peak marked with * in the 2D plot is a persistent impurity identified as 2,4-di-tert-buthylphenol

The resulting chromatograms of the GC-QMS and GC×GC-ToFMS separation are showed in Fig. 1A–B. As can be seen from the 2D plot, more polar pesticides (those containing more heteroatoms) are more retained in the second dimension (*y*-axis). Dieldrin (peak 34) elutes at the bottom of the 2D space, a phenomenon called wrap-around given by the elution of the compounds over the modulation time, and that in this case is not detrimental to the overall separation since it is not coeluting with other compounds of interest.

Figure 1B reports 14 additional pesticides (53 total) which were also validated with the GC×GC-ToFMS method. The possibility for post-targeted analysis, a convenience of the GC×GC-ToFMS, indeed allowed to identify and validate such additional 14 pesticides (I–XIV).

Among these, the analytes captan (peak IX) and folpet (peak X) are thermally unstable pesticides, degrading to 1,2,3,6-tetrahydrophthalimide and phthalimide, respectively. Such degradation conversion for captan and folpet was also evaluated a posteriori, thanks to the non-targeted nature of the GC×GC-ToFMS method. The values resulted on average (*n* = 9) 57% and 47% at the lowest calibration point and 16% and 31% at the highest calibration point, for captan and folpet, respectively. These conversion values were calculated as the ratio between the degradation product and its main form, considering a common characteristic *m/z*, i.e., 79, for 1,2,3,6-tetrahydrophthalimide/captan and 104 phthalimide/folpet.

The GC×GC–MS method was validated in terms of reproducibility (intra- and inter-day), linearity, LOD, and LOQ for the 53 target analytes (Table 1), following Eurachem guidelines [26]. The calibration curves showed a correlation coefficient (*R*^2^) in the range 0.9998–0.9919. Twenty-five of the target analytes were linear within the entire calibration levels ranging from 0.01 (level 1) to 0.15 (level 6) μg.L^−1^; three of the target compounds were linear within the 0.025-0.15 μg.L^−1^ range, while for sixteen of them the linearity range was 0.05–0.15 μg.L^−1^. Eight analytes showed a linearity range between 0.01 and 0.1 μg.L^−1^, and one compound it was between 0.025 and 0.1 μg.L^−1^.

The LOD values ranged from 0.0003 μg.L^−1^ for metolachlor to 0.0022 μg.L^−1^ for ethofumesate and were confirmed by injecting the standard mix at lower concentration (5 ppb, equivalent to 0.0025 μg.L^−1^). In any case, as can be seen in Table 1, the LOQ values were always below the threshold value (0.1 μg.L^−1^). These LOQ values were much lower than those obtained in 1D GC-QMS: the average and median fold increase of the target analytes resulted 8 times lower in GC×GC-ToFMS.

It is worthy to add that a more pragmatic approach for LOQ calculation is reported by SANTE/2020/12830rev1 and SANTE11312/2021 [29, 30]. Here, LOQ is defined as the lowest validated level with sufficient recovery (70–120%) and precision (≤ 20%) and must meet the level of 0.1 ug.L^−1^ in environmental waters [30]. Considering this guidelines, the limit of quantification of the 53 target pesticides resulted 0.01 μg.L^−1^.

An interesting feature of the method validation procedure used in the current study, as described in material and methods, is that the large data set collected throughout several days (25 days) for calibration purposes can be exploited to calculate intra- and inter-day accuracy and precision at all the six concentration levels without requiring any further experimental work.

We report the precision calculated at 0.01 and 0.15 μg.L^−1^, which are the lowest and highest calibration levels, respectively. At the lowest level, target pesticides showed an average CV of 7.6%, in a range from 2.1% (metolachlor) to 15.7% (ethofumesate). At 0.15 μg.L^−1^ level, it was found an average 3% CV, ranging from 0.7% for aldrin to 5.7% for oxadiazon.

The inter-day precision at 0.01 μg.L^−1^ was within the range 3.1–20.4% (for metolachlor and hexachlorobenzene, respectively), with an average value of 10.4; at the highest level, the range of CV% was between 1.7 and 18.8 (for chlorpyrifos-ethyl the lowest value and hexachlorobenzene, respectively), with an average CV% of 6.6.

Trueness was evaluated on spiked blank samples at 0.03 and 0.125 μg.L^−1^, with bias% errors within ± 20%. Only pentachlorobenzene and hexachlorobenzene showed a bias% error of − 23.8 and − 25.9% for the lowest level and − 26.4 and − 30.3% for the highest, respectively. In Electronic Supplementary Material Table S1, the experimental concentrations and CV% of these samples are reported.

The accuracy of the GC×GC–MS method for all the target pesticides was also evaluated using the calibration curve, with relative errors lower than 18%.

In terms of extraction recovery, all the target pesticides showed values over 80% (between 109 and 82%) at 0.01 μg.L^−1^. Such results are illustrated in Electronic Supplementary Material Figure S1, which also reports the extraction recovery obtained at 0.15 μg.L^−1^.

### Application to real-world samples

Thirty-four extracts from surface and groundwater samples were then injected in both GC systems, using the developed GC×GC-ToFMS and the routine GC-QMS methods for the determination of the validated pesticides.

**Fig. 2:**
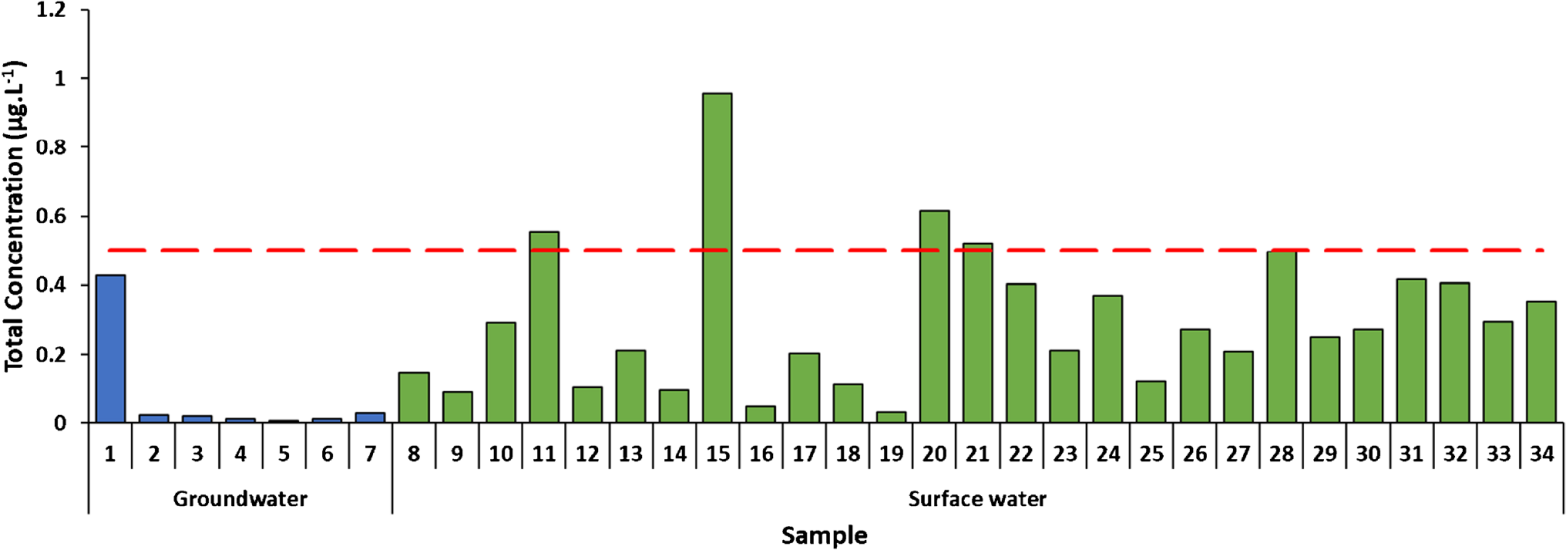
Total pesticides concentration of samples by GC×GC-MS analysis. The red line represents the threshold value of the total concentration (0.5 μg.L^−1^)

Figure 2 reports the pesticides’ total concentration, and it shows that most of the samples contain a low amount of pesticides and only 4 of them exceeded the threshold value (0.5 μg.L^−1^). However, regarding the quantities of single compounds, it was found that at least one pesticide exceeded the limit in 14 samples. The quantitative results in the samples are found in Electronic Supplementary Material Figure S2.

As one can expect, groundwater samples are less prone to pesticide contamination than the surface water samples; in fact, their average concentrations are 0.076 μg.L^−1^ and 0.298 μg.L^−1^, respectively.

**Fig. 3:**
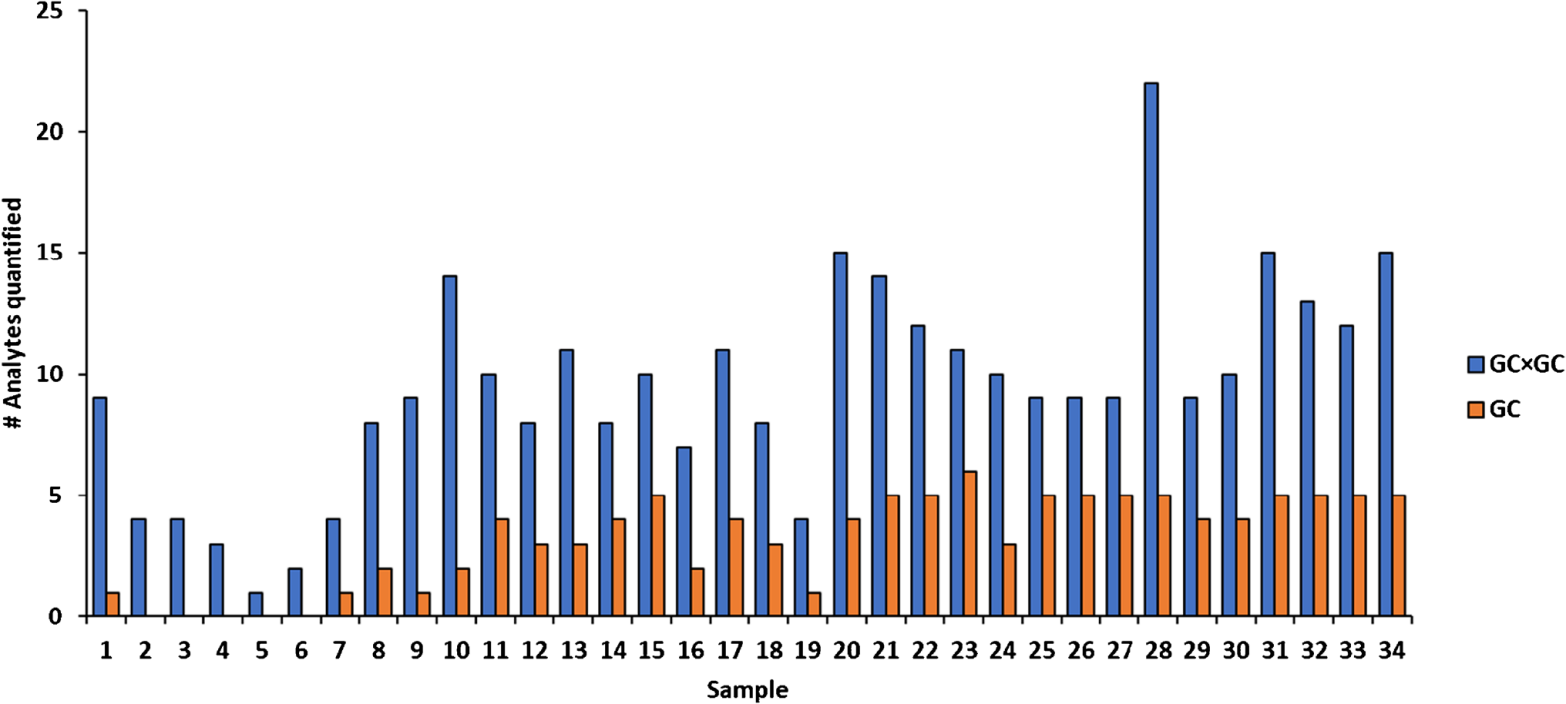
Number of pesticides quantified by GC-QMS and GC×GC-ToFMS in water samples

Figure 3 shows the comparison of the number of analytes quantified between the two methods. With GC×GC, more pesticides were detected and quantified due to the higher sensitivity.

Noteworthy, with the GC×GC-ToFMS system, most of the quantifiable analytes were detected at lower concentration with respect to GC-QMS (Figure S3). The disagreement can be explained by the fact that some pesticides might coelute with other interferences. Indeed, the coelutions that are resolved in the ^2^D of a multidimensional system would otherwise give a quantitative overestimation in 1D separations.

Here, it is shown that 44% of all analytes quantified in the various samples in both GC×GC and GC method are found to have comparable concentration between the two techniques (within ± 20% error), while 48% find a higher concentration in GC-MS and only 8% higher in two-dimensional technique. Also, most of the target analytes were below the LOQ, thus not reported in Figure S3.

**Fig. 4:**
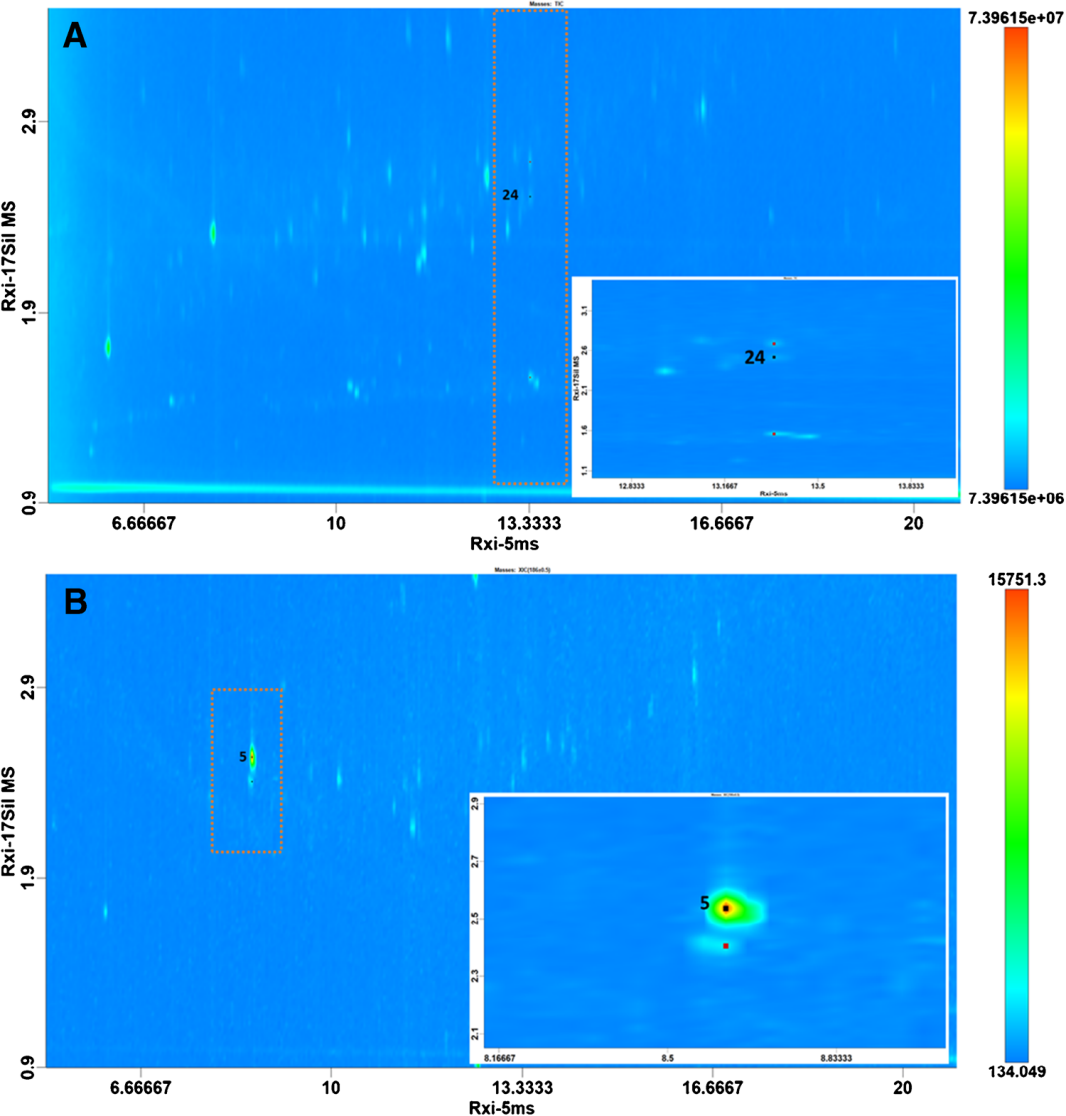
**A–B** Bidimensional plots of a surface water extract highlighting two examples of chromatographic coelutions which are resolved in the second dimension. **A** 2D plot (TIC) in which the target analyte metolachlor (black dot) is resolved from two potential interferences (red dots). **B** 2D plot (186 *m/z*) in which the target analyte desethylterbuthylazine (black dot) is resolved from one interference (red dot)

A representative resolved peak pairs by GC×GC that alternatively would be coeluted in 1D separation is shown in Fig. 4A. In the inset, the target analyte metolachlor (black dot) is separated in the ^2^D from a less retained and a more retained compound (black dots). More importantly, in Fig. 4B, it is visualized the GC×GC extracted ion chromatogram of a water extract, referred to 186 *m/z* (i.e., the quantifier ion of target analyte desethylterbuthylazine (#5)), which is contributing to the overestimation in 1D GC.

## Conclusions

A method involving SPE purification followed by GC×GC-ToFMS analysis was herein evaluated for the determination of 53 pesticides in environmental samples and specifically surface and groundwater. Method validation in terms of linearity, precision, and accuracy showed satisfactory results. The limits of quantifications ranged from 0.0011 to 0.0072 μg.L^−1^, values well below the regulated 0.1 μg.L^−1^ limit per single analyte. According to the recent SANTE guidelines, LOQ values with a CV% (precision) ≤ 20% and a recovery between 70 and 120% were 0.01 μg.L^−1^.

The validated methodology was applied to 34 real-world samples, and the quantitative results were compared with a routine GC-QMS method. Worthy to note is that in 54 determinations (48% of the total positive cases), it was observed an overestimation (over ± 20% error) using the one-dimensional method which can be caused by its lower selectivity, compared to the GC×GC-ToFMS.

Beside the higher sensitivity (eightfold on LOQ values) and faster analysis time (40%), another important advantage of the developed GC×GC-ToFMS is its non-targeted nature, which can allow a posteriori verification of the presence of not yet regulated pesticides in environmental samples.

It can be said that the same methodology fits or can be undoubtedly adapted to environmental samples of different nature (e.g., soil, air) for pesticides determination, thanks to its non-targeted feature.

However, an important step that was not explored in detail in our research is the sample preparation, and future research will be devoted to further explore, miniaturize, and develop the extraction techniques to extend the target analytes coverage.

## Supplementary Information

Below is the link to the electronic supplementary material.

Supplementary file1 (DOCX 2050 KB)

## References

[1] de Souza RM, Seibert D, Quesada HB, de Jesus BF, Fagundes-Klen MR, Bergamasco R (2020). Occurrence, impacts and general aspects of pesticides in surface water: a review. Process Saf Environ Prot.

[2] (2019) European Commission. In: https://food.ec.europa.eu/plants/pesticides/eu-pesticides-database_en. Accessed 7/11/2022.

[3] Hassaan MA, el Nemr A (2020). Pesticides pollution: classifications, human health impact, extraction and treatment techniques. Egypt J Aquat Res.

[4] Delcour I, Spanoghe P, Uyttendaele M (2015). Literature review: impact of climate change on pesticide use. Food Res Int.

[5] Ecobichon DJ (2001). Pesticide use in developing countries. Toxicology.

[6] Stockholm Convention. In: http://chm.pops.int/Home/tabid/2121/Default.aspx. Accessed 7/11/2022.

[7] Rajmohan KS, Chandrasekaran R, Varjani S (2020). A review on occurrence of pesticides in environment and current technologies for their remediation and management. Indian J Microbiol.

[8] Syafrudin M, Kristanti RA, Yuniarto A, Hadibarata T, Rhee J, Al-onazi WA, Algarni TS, Almarri AH, Al-Mohaimeed AM (2021). Pesticides in drinking water—a review. Int J Environ Res Public Health.

[9] Aydinalp C, Porca MM (2004). The effects of pesticides in water resources. J Cent Eur Agric.

[10] Directive 2000/60/EC of the European Parliament and of the Council of 23 October 2000 establishing a framework for community action in the field of water policy. https://eur-lex.europa.eu/legal-content/en/ALL/?uri=CELEX%3A32000L0060. Accessed 17 Apr 2023.

[11] Directive (EU) 2020/2184 of the European Parliament and of the Council of 16 December 2020 on the quality of water intended for human consumption. https://eur-lex.europa.eu/eli/dir/2020/2184/oj. Accessed 17 Apr 2023.

[12] Directive 2006/118/EC of the European Parliament and of the Council of 12 December 2006 on the protection of groundwater against pollution and deterioration. https://eur-lex.europa.eu/legal-content/EN/TXT/?uri=celex%3A32006L0118. Accessed 17 Apr 2023.

[13] Nasiri M, Ahmadzadeh H, Amiri A (2020). Sample preparation and extraction methods for pesticides in aquatic environments: a review. TrAC Trends Anal Chem.

[14] Lucentini L, Patriarca M (2019) Analytical methods for water intended for human consumption according to the Italian Legislative Decree 31/2001. Chemical methods. https://www.iss.it/documents/20126/45616/19_7_web.pdf/8363058b-a214-f255-4ff0-ee04e0a2cfd7?t=1581095853354. Accessed 17 Apr 2023.

[15] Pan H-J, Ho W-H (2004). Determination of fungicides in water using liquid phase microextraction and gas chromatography with electron capture detection. Anal Chim Acta.

[16] Sun S, Li Y, Lv P, Punamiya P, Sarkar D, Dan Y, Ma J, Zheng Y. Determination of prometryn in vetiver grass and water using gas chromatography–nitrogen chemiluminescence detection. J Chromatogr Sci. 2015; bmv108. 10.1093/chromsci/bmv108. 10.1093/chromsci/bmv108 26250891

[17] Pico Y, Alfarhan AH, Barcelo D (2020). How recent innovations in gas chromatography-mass spectrometry have improved pesticide residue determination: an alternative technique to be in your radar. TrAC Trends Anal Chem.

[18] Zanella D, Focant J, Franchina FA (2021). 30^th^ anniversary of comprehensive two-dimensional gas chromatography: latest advances. Anal Sci Adv.

[19] Muscalu AM, Górecki T (2018). Comprehensive two-dimensional gas chromatography in environmental analysis. TrAC Trends Anal Chem.

[20] Engel E, Ratel J, Blinet P, Chin S-T, Rose G, Marriott PJ (2013). Benchmarking of candidate detectors for multiresidue analysis of pesticides by comprehensive two-dimensional gas chromatography. J Chromatogr A.

[21] Khummueng W, Trenerry C, Rose G, Marriott PJ (2006). Application of comprehensive two-dimensional gas chromatography with nitrogen-selective detection for the analysis of fungicide residues in vegetable samples. J Chromatogr A.

[22] Muscalu AM, Reiner EJ, Liss SN, Chen T, Ladwig G, Morse D (2011). A routine accredited method for the analysis of polychlorinated biphenyls, organochlorine pesticides, chlorobenzenes and screening of other halogenated organics in soil, sediment and sludge by GCxGC-μECD. Anal Bioanal Chem.

[23] Arena A, Zoccali M, Mondello L, Tranchida PQ (2022). A method for the determination of 70 pesticides in extra virgin olive oil based on a limited-volume solvent extraction step prior to comprehensive two-dimensional gas chromatography-tandem mass spectrometry. Anal Bioanal Chem.

[24] Mazza FC, de Souza Sampaio NA, von Mühlen C (2022). Hyperspeed method for analyzing organochloride pesticides in sediments using two-dimensional gas chromatography–time-of-flight mass spectrometry. Anal Bioanal Chem.

[25] Tranchida PQ, Franchina FA, Zoccali M, Bonaccorsi I, Cacciola F, Mondello L (2013). A direct sensitivity comparison between flow-modulated comprehensive 2D and 1D GC in untargeted and targeted MS-based experiments. J Sep Sci.

[26] Magnusson B, Ornemark U. Eurachem Guide: the fitness for purpose of analytical methods – a laboratory guide to method validation and related topics, Second edition; 2014. Available from http://www.eurachem.org.

[27] Alladio E, Amante E, Bozzolino C, Seganti F, Salomone A, Vincenti M, Desharnais B (2020). Effective validation of chromatographic analytical methods: the illustrative case of androgenic steroids. Talanta.

[28] Alladio E, Amante E, Bozzolino C, Seganti F, Salomone A, Vincenti M, Desharnais B (2020). Experimental and statistical protocol for the effective validation of chromatographic analytical methods. MethodsX.

[29] Guidance SANTE 11312/2021 on analytical quality control and method validation procedures for pesticide residues analysis in food and feed. https://food.ec.europa.eu/system/files/2022-02/pesticides_mrl_guidelines_wrkdoc_2021-11312.pdf. Accessed 17 Apr 2023.

[30] Guidance Document SANTE 2020/12830, Rev.1 on pesticide analytical methods for risk assessment and post-approval control and monitoring purposes. https://food.ec.europa.eu/system/files/2021-03/pesticides_ppp_app-proc_guide_res_mrl-guidelines-2020-12830.pdf. Accessed 17 Apr 2023.

