# Supplementary material for: Development and validation of a GC × GC-ToFMS method for the quantification of pesticides in environmental waters
Source: Anal Bioanal Chem. 2023 Apr 24;415(18):4545–55. doi: 10.1007/s00216-023-04686-8 (PMC10328859; doi:10.1007/s00216-023-04686-8)

***Supplementary Table S1.*** *Fortification standards’ figure-of-merits: experimental concentrations and CV% of target analytes at 0.03 and 0.125 μg.L^-1^ levels.*

| **Peak Number** | **Analytes** | **Experimental concentration at 0.03 μg.L^-1^ (μg.L^-1^)** | **Experimental concentration at 0.125 μg.L^-1^ (μg.L^-1^)** | **CV% at 0.03 μg.L^-1^** | **CV% at 0.125 μg.L^-1^** |
| --- | --- | --- | --- | --- | --- |
| 1 | Benzene, pentachloro- | 45.7 | 183.9 | 13.0 | 12.4 |
| 2 | Molinate | 51.0 | 215.1 | 5.7 | 5.0 |
| 3 | Desethylatrazine | 56.6 | 230.4 | 9.8 | 10.2 |
| 4 | Trifluralin | 51.5 | 211.1 | 13.6 | 11.2 |
| 5 | Desethylterbuthylazine | 57.0 | 229.6 | 8.4 | 8.4 |
| 6 | α-BHC | 60.5 | 245.4 | 8.1 | 6.6 |
| 7 | Benzene, hexachloro- | 44.5 | 174.3 | 12.2 | 7.7 |
| 8 | Dimethoate | 69.0 | 269.5 | 17.7 | 2.1 |
| 9 | Simazine | 50.9 | 208.2 | 13.3 | 13.9 |
| 10 | Atrazine | 55.1 | 223.9 | 11.6 | 10.1 |
| 11 | β-BHC | 56.6 | 229.7 | 8.5 | 7.0 |
| 12 | Lindane | 56.6 | 230.3 | 8.2 | 7.5 |
| 13 | Terbuthylazine | 56.7 | 225.2 | 10.1 | 10.6 |
| 14 | Propyzamide | 59.3 | 234.4 | 9.2 | 8.8 |
| 15 | δ-BHC | 59.0 | 238.0 | 5.5 | 7.4 |
| 16 | Caffeine | 47.4 | 209.1 | 18.3 | 13.6 |
| 17 | Propanil | 58.5 | 252.8 | 12.6 | 5.4 |
| 18 | Dimethenamid | 58.6 | 239.1 | 8.9 | 6.2 |
| 19 | Chlorpyrifos-methyl | 61.9 | 244.0 | 14.4 | 4.2 |
| 20 | Alachlor | 63.3 | 258.9 | 4.9 | 4.0 |
| 21 | Heptachlor | 61.9 | 231.7 | 5.2 | 3.8 |
| 22 | Terbutryn | 56.6 | 230.4 | 9.8 | 10.2 |
| 23 | Aldrin | 53.4 | 230.4 | 4.2 | 1.3 |
| 24 | Metolachlor | 59.4 | 242.9 | 5.9 | 5.4 |
| 25 | Chlorpyrifos-ethyl | 60.2 | 250.8 | 10.1 | 1.9 |
| 26 | Isodrin | 52.9 | 209.6 | 10.4 | 8.6 |
| 27 | Metazachlor | 60.0 | 256.4 | 13.4 | 4.2 |
| 28 | Pendimethalin | 60.9 | 238.4 | 9.4 | 9.2 |
| 29 | Heptachlor epoxide | 58.6 | 240.3 | 0.3 | 1.1 |
| 30 | Chlorfenvinphos | 57.0 | 242.9 | 7.2 | 4.6 |
| 31 | α-Endosulfan | 56.6 | 230.4 | 9.8 | 10.2 |
| 32 | p,p'-DDE | 54.4 | 216.9 | 11.0 | 11.8 |
| 33 | Oxadiazon | 61.0 | 241.8 | 14.2 | 13.6 |
| 34 | Dieldrin | 57.1 | 245.7 | 4.7 | 2.9 |
| 35 | Endrin | 64.9 | 258.0 | 4.0 | 1.3 |
| 36 | β-Endosulfan | 59.7 | 244.6 | 18.8 | 4.1 |
| 37 | p,p'-DDD | 56.4 | 225.9 | 10.4 | 10.3 |
| 38 | o,p'-DDT | 54.9 | 220.8 | 9.0 | 11.1 |
| 39 | p,p'-DDT | 56.8 | 231.3 | 7.1 | 10.0 |
| I | Prometon | 68.5 | 263.7 | 14.5 | 3.2 |
| II | Vinclozolin | 61.5 | 252.2 | 5.8 | 7.0 |
| III | Ametryn | 57.3 | 264.1 | 7.1 | 4.6 |
| IV | Prometryn | 57.9 | 236.5 | 10.4 | 10.9 |
| V | Ethofumesate | 59.1 | 243.8 | 9.7 | 2.2 |
| VI | Malathion | 59.9 | 242.4 | 4.4 | 6.3 |
| VII | Bladex | 62.8 | 277.7 | 10.5 | 3.6 |
| VIII | Flufenacet | 62.0 | 275.2 | 4.2 | 3.4 |
| IX | Captan | 61.3 | 279.6 | 25.8 | 11.7 |
| X | Folpet | 53.2 | 254.5 | 12.3 | 8.7 |
| XI | Procymidone | 58.3 | 248.6 | 6.2 | 2.4 |
| XII | trans-Chlordane | 50.8 | 201.7 | 8.3 | 7.2 |
| XIII | cis-Chlordane | 54.1 | 215.1 | 8.1 | 9.3 |
| XIV | Endosulfan sulfate | 60.8 | 251.1 | 3.8 | 5.8 |


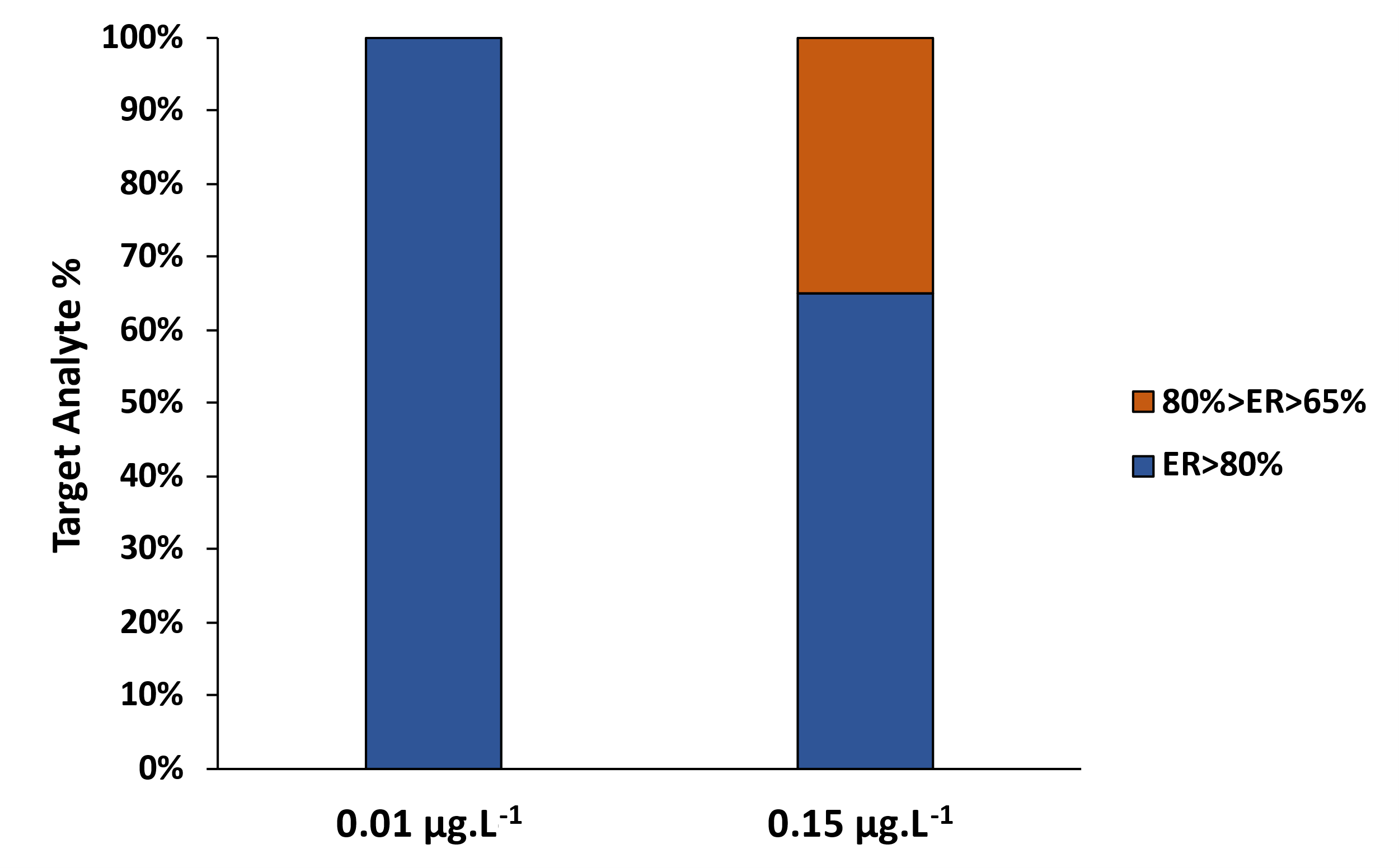


***Supplementary Figure S1.*** *Trend of analytes extraction recovery at 0.01 and 0.15 μg.L^-1^.*

***Supplementary Figure S2.*** *Detectable compounds in the 37 groundwater (GW, in blue) and surface water (SW, in green) samples by GC×GC-ToFMS. Compounds with * or # were detected, but not quantified according to Eurachem or SANCO/SANTE limits, e.g. <LOQ_EURACHEM_ and <LOQ_SANCO/SANTE_, respectively.*


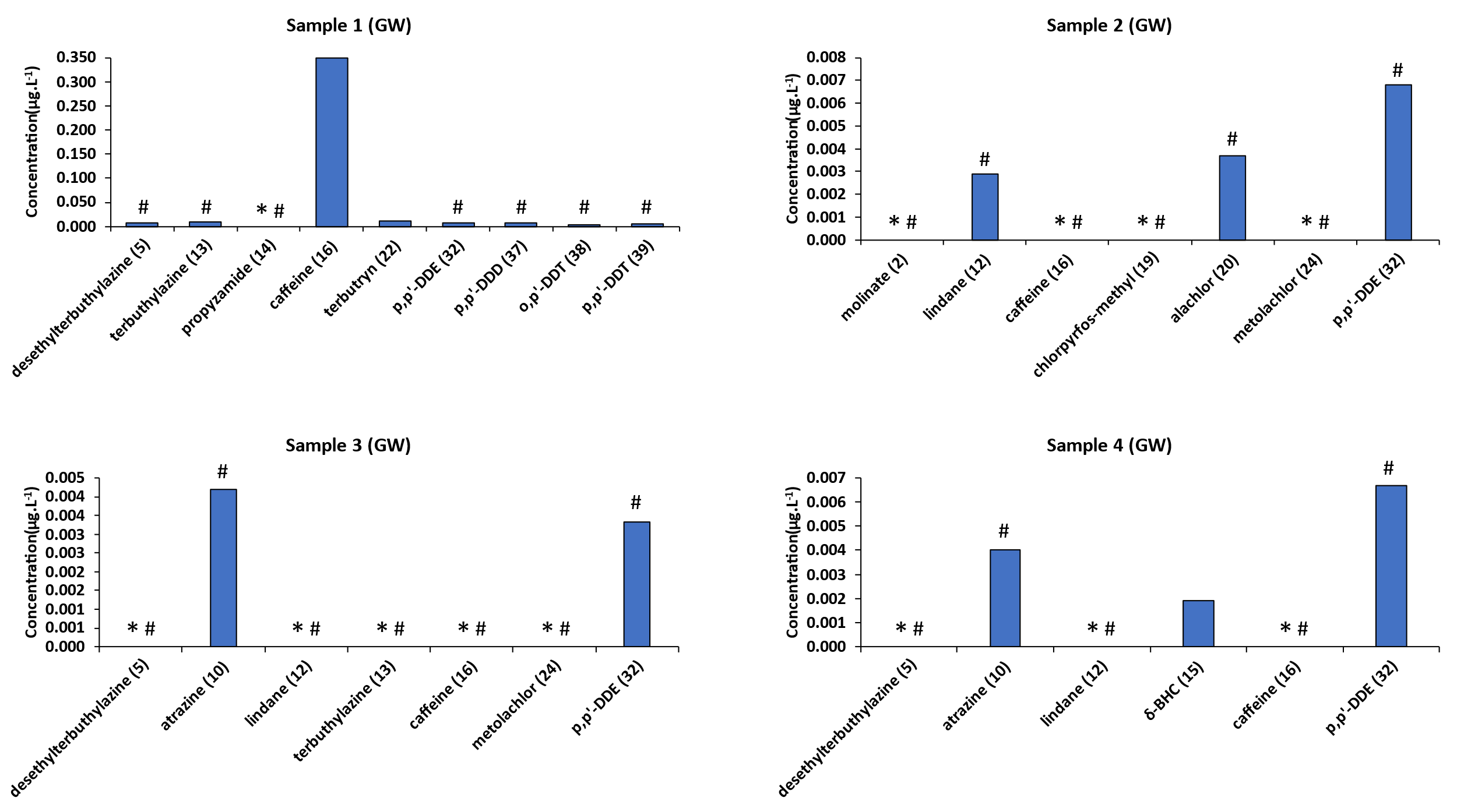


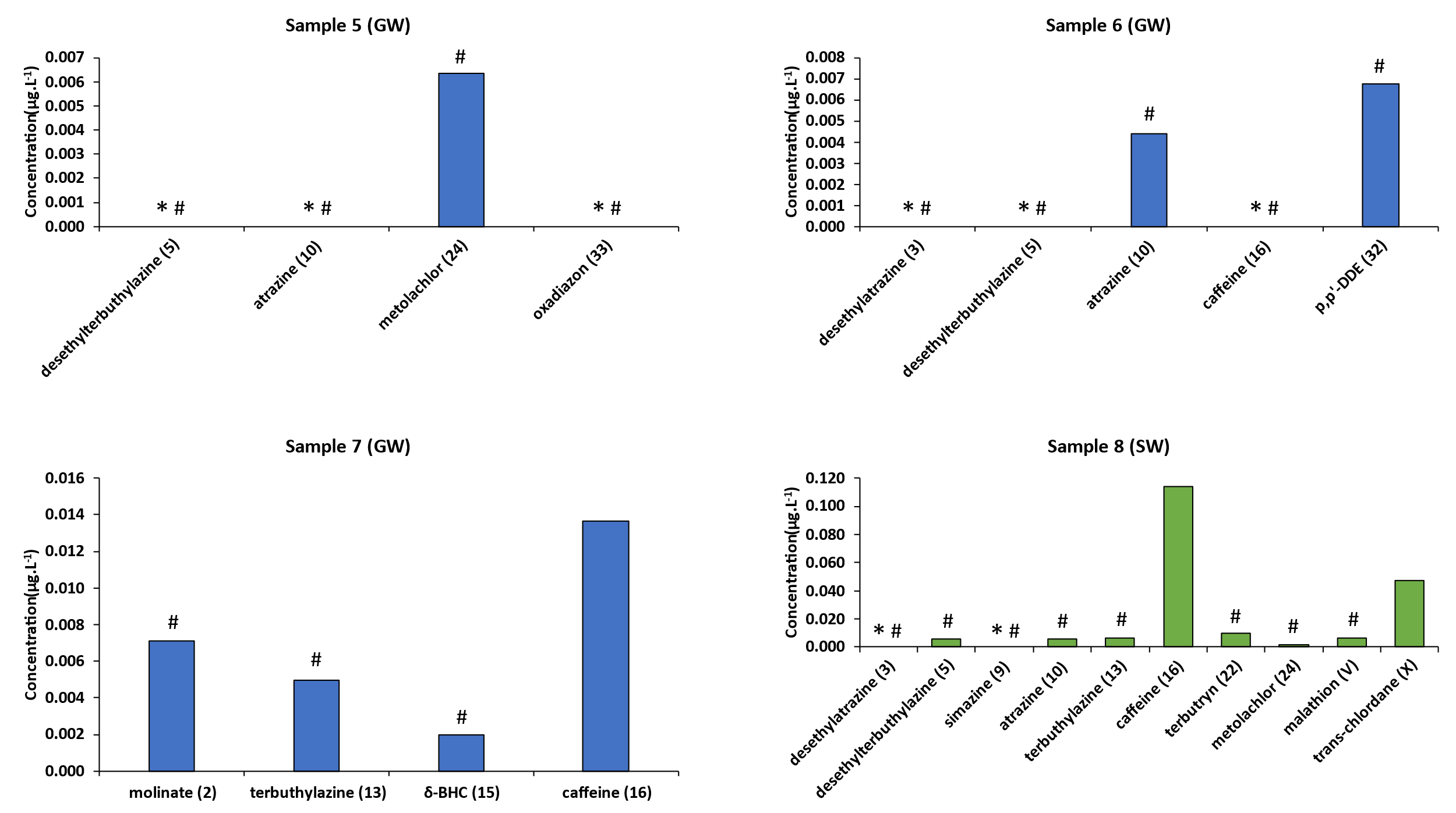


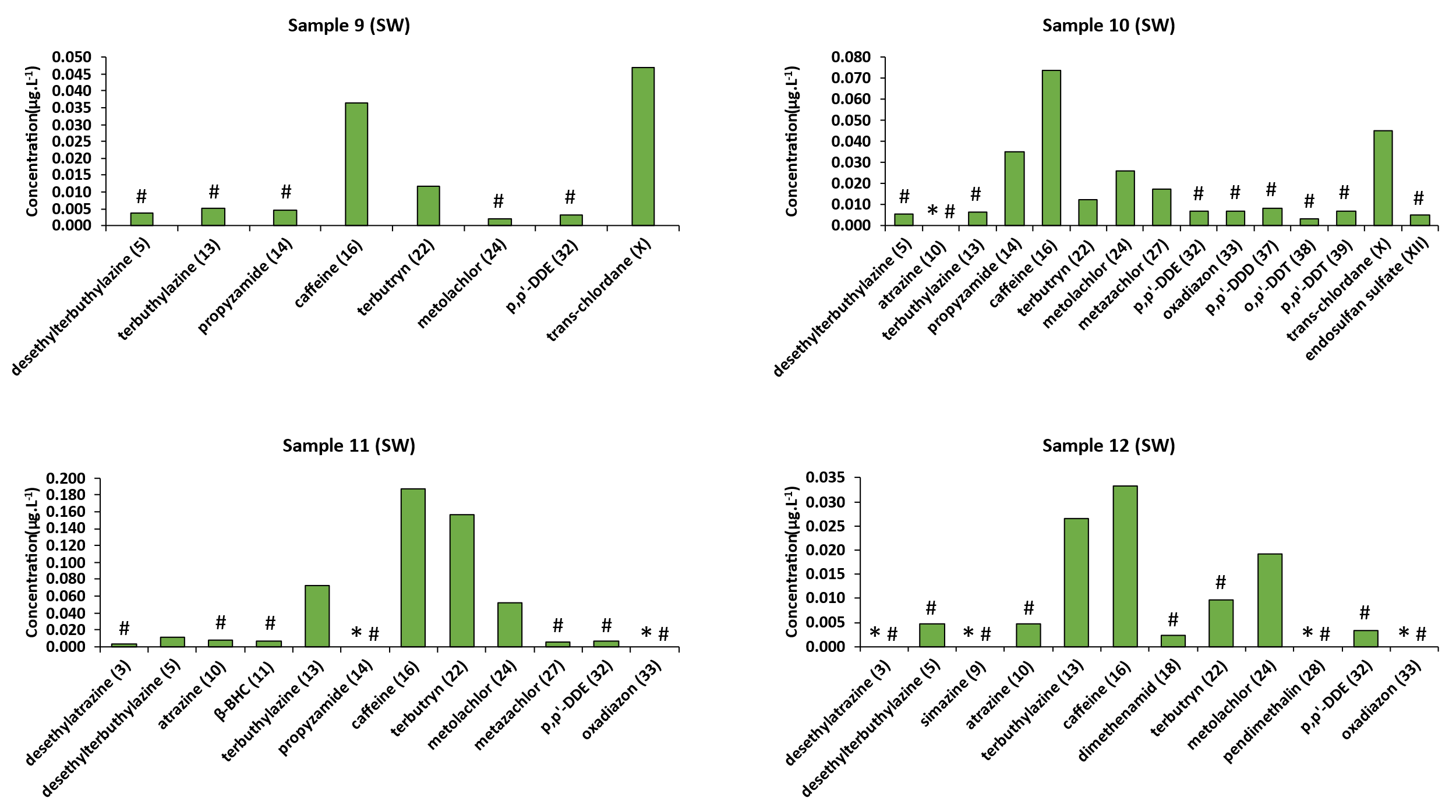


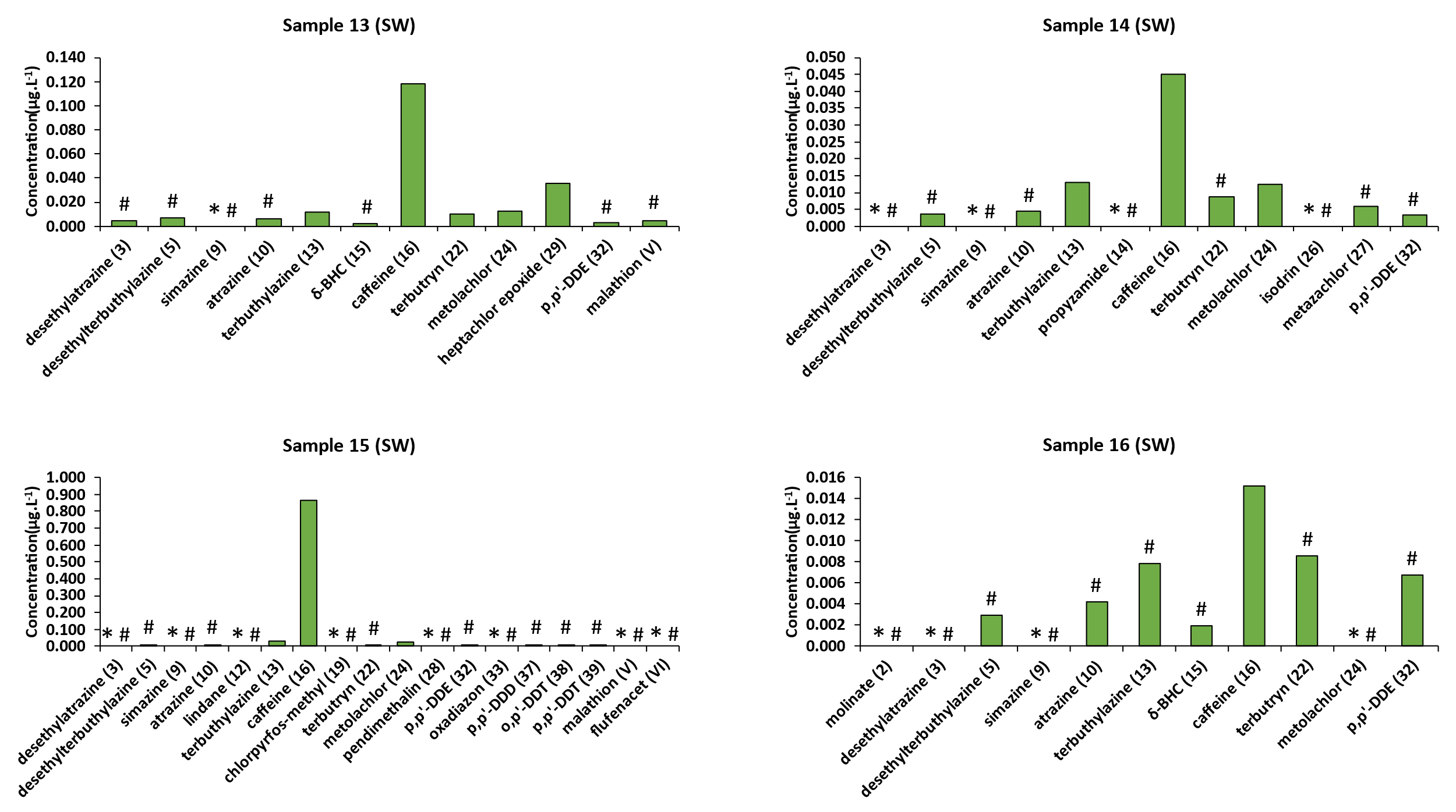


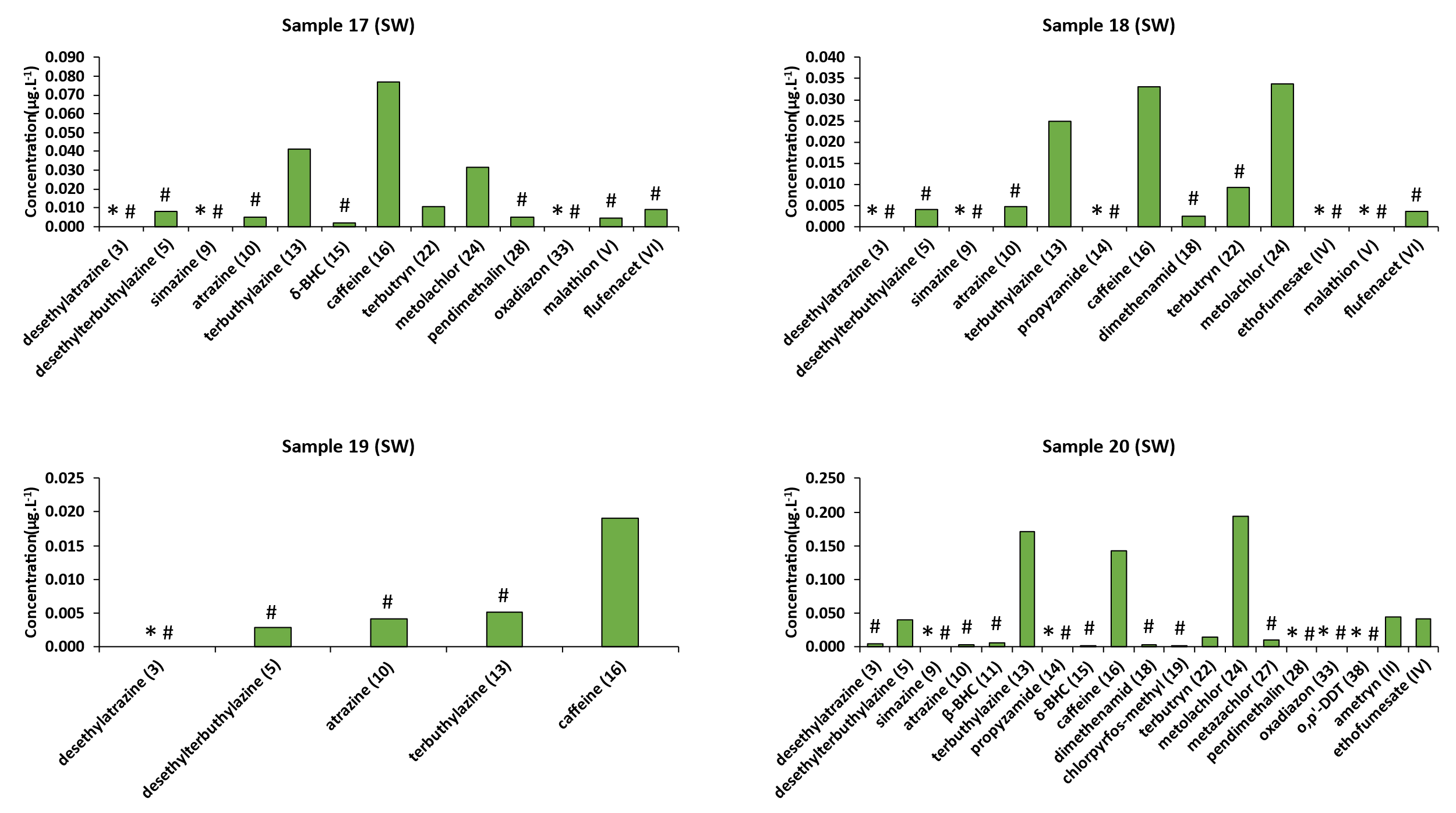


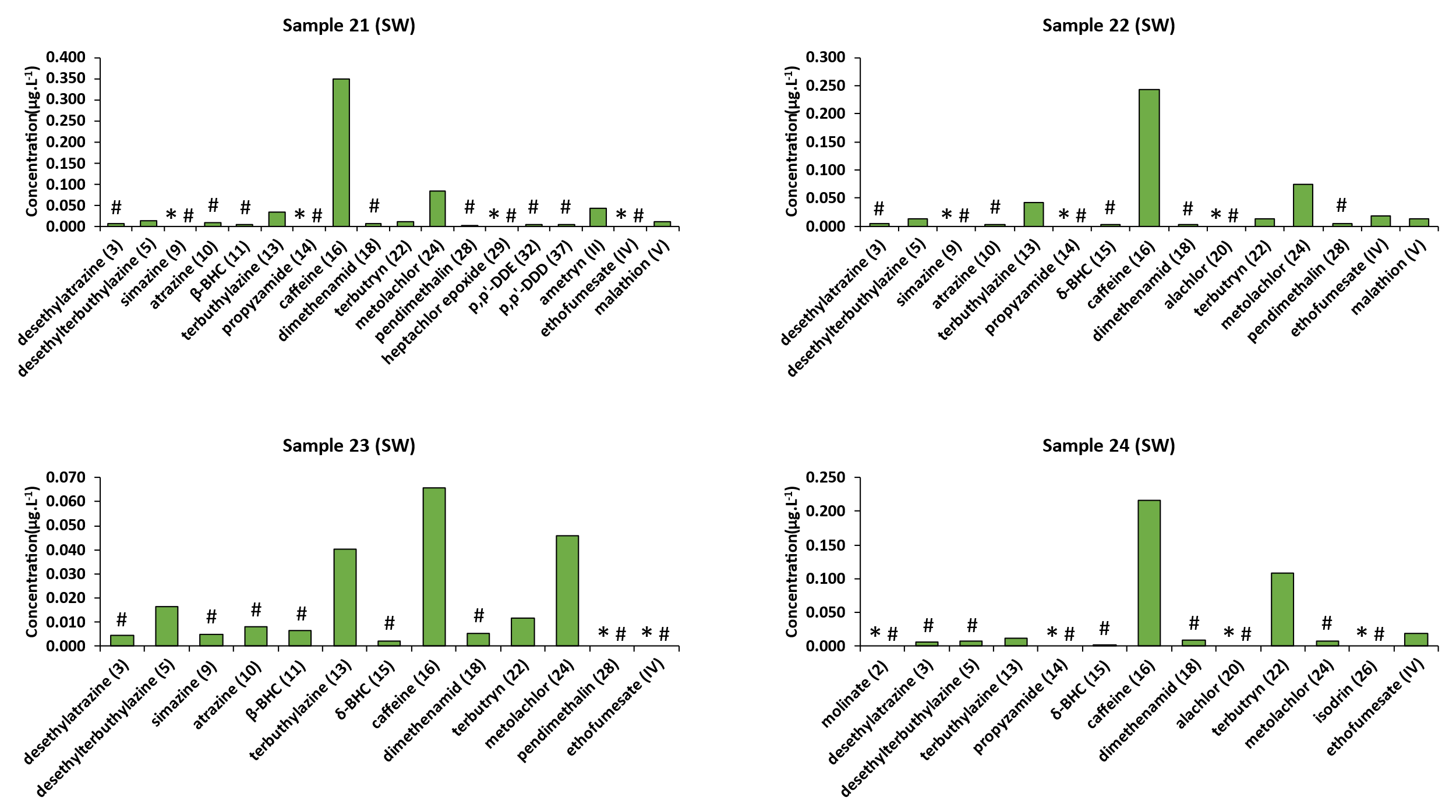


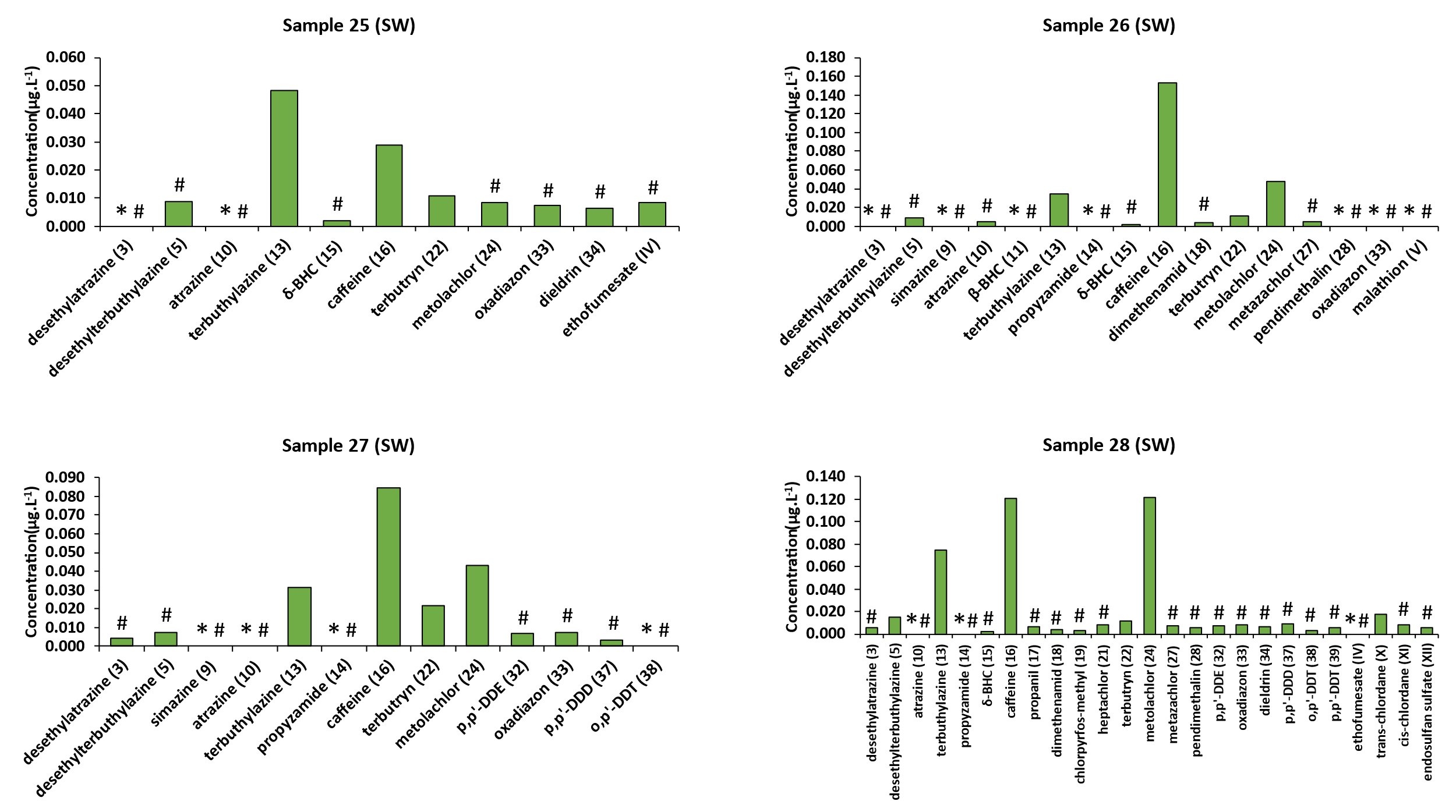


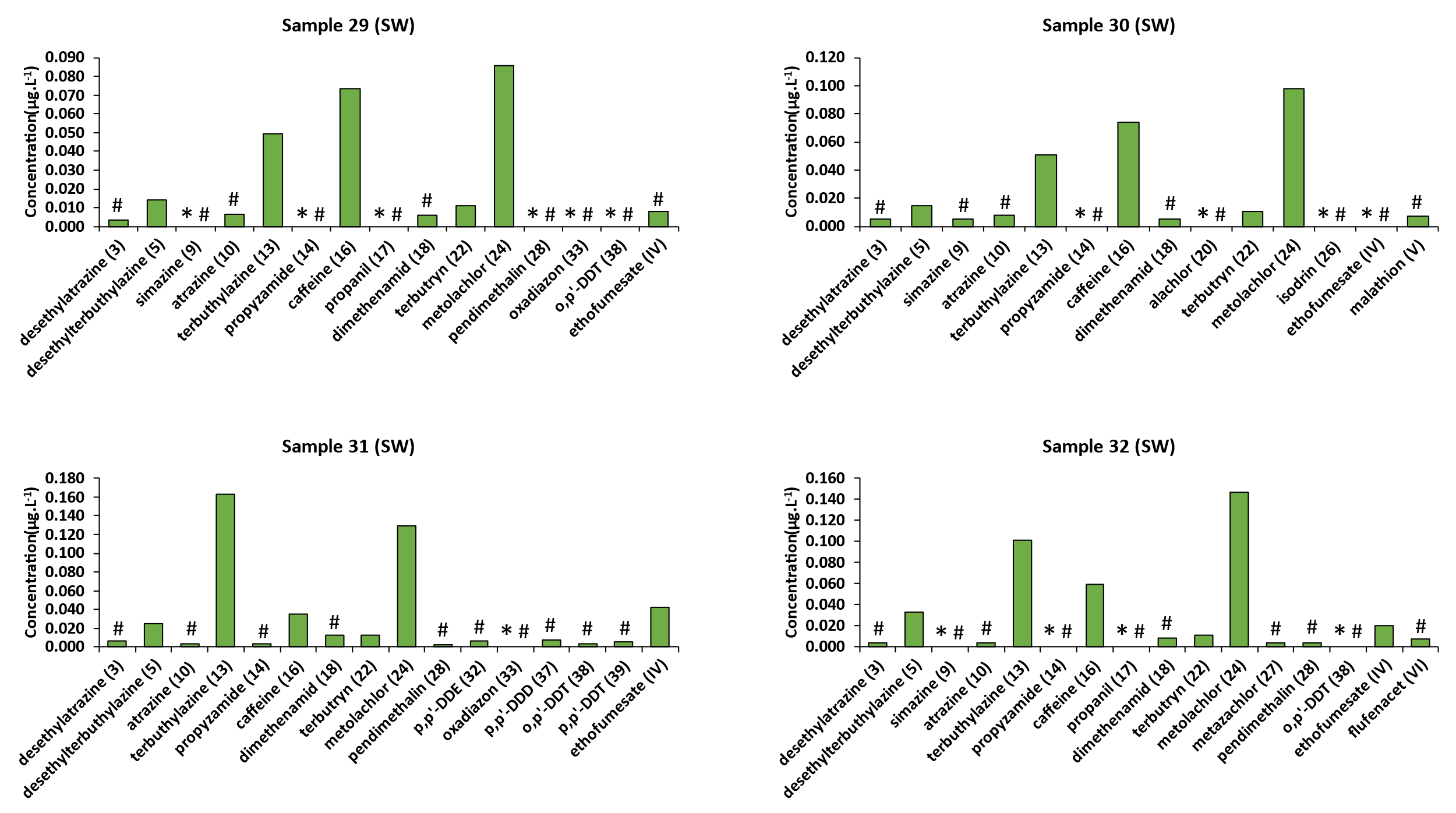


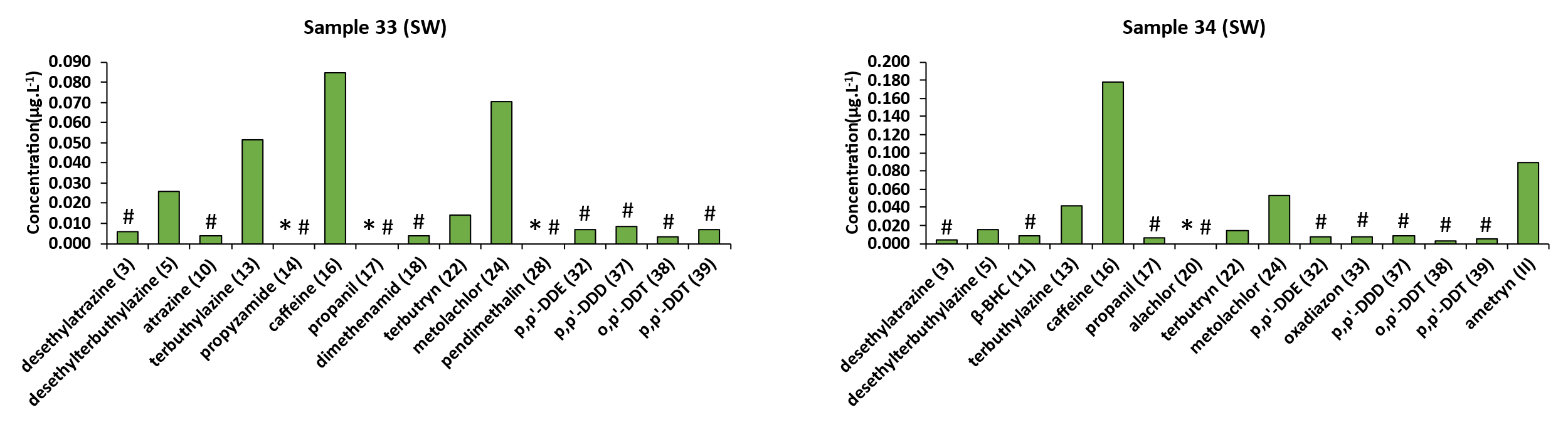


***Supplementary Figure S3.*** *Analyte quantitation results: comparative view between GC-QMS and GC×GC-ToFMS.*


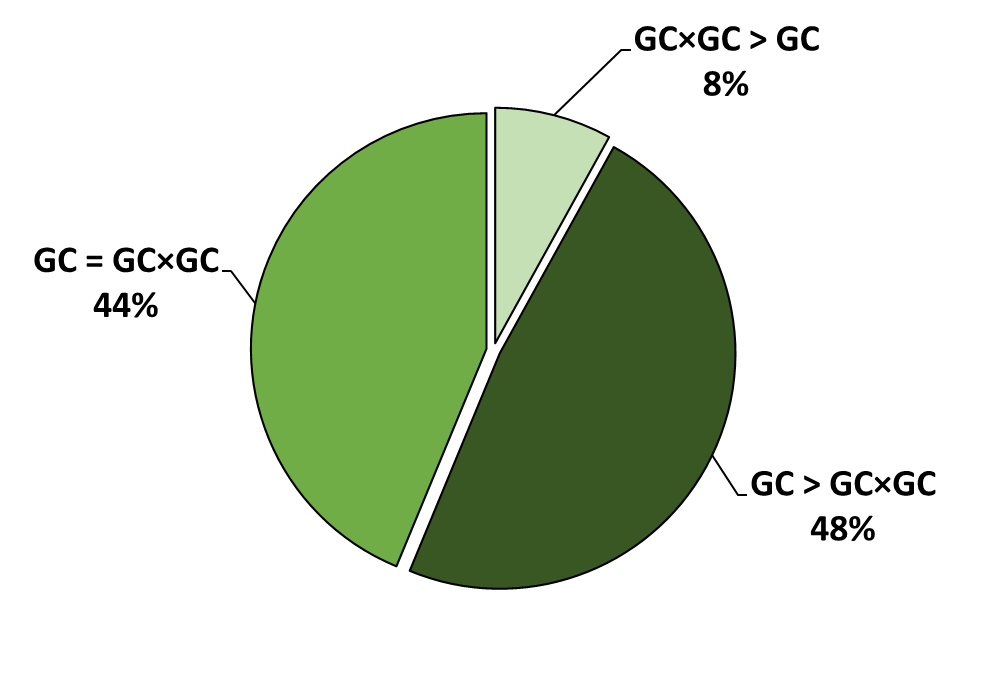

Supplement: Supplementary file 1 — Supplementary file1 (DOCX 2050 KB) [file 216_2023_4686_MOESM1_ESM.docx]
